# AI Systems and Respect for Human Autonomy

**DOI:** 10.3389/frai.2021.705164

**Published:** 2021-10-26

**Authors:** Arto Laitinen, Otto Sahlgren

**Affiliations:** Faculty of Social Sciences, Tampere University, Tampere, Finland

**Keywords:** autonomy, self-determination, respect, artificial intelligence, human-centered AI, ought to be, sociotechnical base

## Abstract

This study concerns the sociotechnical bases of human autonomy. Drawing on recent literature on AI ethics, philosophical literature on dimensions of autonomy, and on independent philosophical scrutiny, we first propose a multi-dimensional model of human autonomy and then discuss how AI systems can support or hinder human autonomy. What emerges is a philosophically motivated picture of autonomy and of the normative requirements personal autonomy poses in the context of algorithmic systems. Ranging from consent to data collection and processing, to computational tasks and interface design, to institutional and societal considerations, various aspects related to sociotechnical systems must be accounted for in order to get the full picture of potential effects of AI systems on human autonomy. It is clear how human agents can, for example, via coercion or manipulation, hinder each other’s autonomy, or how they can respect each other’s autonomy. AI systems can promote or hinder human autonomy, but can they literally respect or disrespect a person’s autonomy? We argue for a philosophical view according to which AI systems—while not moral agents or bearers of duties, and unable to literally respect or disrespect—are governed by so-called “ought-to-be norms.” This explains the normativity at stake with AI systems. The responsible people (designers, users, etc.) have duties and ought-to-do norms, which correspond to these ought-to-be norms.

## Introduction

It is relatively clear that AI technology can make a difference to the conditions of human autonomy, and it would be surprising if the difference it makes could not be negative or positive. The more ubiquitous AI technology becomes, the more important it is to understand its ethical effects. And as, indeed, “digital technologies now mediate most human experience” (Calvo et al., 2020), AI technology is already quite ubiquitous. From decision and recommender systems to self-tracking technologies and autonomous vehicles, AI systems can have more or less subtle, but nonetheless, far-reaching effects on how humans deliberate and behave.

It is not surprising that human autonomy has been recognized as integral for human-centered design and the use of AI—a central value in its own right, warranting protection from the possible ill effects of technology (Anderson and Rainie, 2018). Indeed, protecting autonomy can be regarded as a separate, valuable aim in algorithmic decision systems, conceptually distinct from other values, such as fairness. Human autonomy is, in this sense, an ethical value in its own right, grounding a right to self-determination and corresponding duties of other agents (e.g., non-interference and prohibition of subordination). While AI ethics is not solely about autonomy, autonomy is relevant in surprisingly many ways, not least because it is significantly intertwined with other values, such as privacy (Lanzing, 2016; Lanzing, 2019), transparency (Rubel et al., 2021), and human dignity (Riley and Bos, 2021).

Existing research has identified several autonomy-related issues with AI systems, including ways in which different systems may undermine or constrain human autonomy. AI technologies are already used to dynamically personalize individual’s choice environments, to paternalistically nudge, deceive, and even manipulate behavior in unprecedented manners (Yeung, 2017; Susser et al., 2019). Increasing deference to algorithmic systems in various decision-making processes raises the question of whether user’s choices can be regarded as authentic, and whether this tendency will impoverish our capacity for self-determination (see Danaher, 2018). Furthermore, one might ask, given that algorithmic decision-making practices are based on past and group-level data, can they truly be sensitive to a person’s capacity to author their own life (see Kaminski, 2018)?

This article discusses these issues among various other effects AI systems can have on *human autonomy*. We begin with a discussion of the human aspects of autonomy in a theoretical way outside the context of AI and then discuss how AI can be a burden to human autonomy. It is naturally good to characterize autonomy before assessing potential effects on autonomy, but more importantly, working out a multidimensional model of autonomy provides a list of questions: how can AI conceivably, or based on the literature, affect this or that dimension of autonomy (including such indirect effects as the role of cultural resources in autonomy)? We focus mainly on decision systems and recommenders, although we expect our conclusions to generalize in relevant ways to other technologies as well. We agree with Rubel et al. (2020) who argue that “a complete picture of the moral salience of algorithmic systems requires understanding algorithms as they relate to agency, autonomy, and respect for persons.” Our method is philosophical reflection and model-building guided by existing literature on AI ethics and philosophy of human autonomy.

The study will consider what the right to self-determination implies for the ethics of designing and using AI systems. We approach the question in two ways: first, by studying what respect for autonomy requires from other moral agents, and hypothesizing that analogous normative requirements apply for AI systems, and second, by studying other preconditions of autonomy and seeing how AI systems can support or prevent those preconditions. What emerges is a hopefully nuanced and philosophically motivated picture of the normative requirements personal autonomy poses in the context of algorithmic systems, ranging from user-level to institutional and societal considerations. To the best of our knowledge, similarly comprehensive accounts of personal autonomy as it relates to AI have not been offered in the literature.

In more detail, the article is structured as follows. In *Aspects of Autonomy: Capacities, Requirements, Respect, Self-Respect, Exercise, and Resources*, we discuss different aspects of autonomy and propose a revisable, multi-dimensional model of constituent aspects and preconditions of human autonomy, which will structure the discussion throughout. The dimensions of autonomy considered here include 1) potential and developed capacities for self-determination, 2) normative requirements or duties to respect and support human autonomy, 3) relational aspects of autonomy, most notably recognition and respect from others, 4) self-respect, and 5) exercise of one’s autonomy. In addition, 6) various material, economic, legal, cultural, and informational resources can be understood as comprising necessary conditions for autonomy, although they are not in themselves constitutive of autonomy. In *Ethics Concerning Non-Agents: Ought-To-Be Norms and Quasi-Respect*, we ask whether AI systems can be said to literally respect autonomy. According to our account, while AI systems are not moral agents (at least currently), and thus cannot have duties or literally respect or disrespect, they are governed by so-called “ought-to-be norms”. In *Interpersonal Disrespect and Violations by AI Systems*, we zoom in on the violations of autonomy on behalf of other humans and corresponding violations by AI systems. We list many such violations, but we limit the more detailed discussion to three issues that have received special attention in the literature on decision and recommender systems: coercion and manipulation in the context of AI systems, cognitive heteronomy, and direct misrecognition of human autonomy. In *The Prerequisites and Sociotechnical Bases of Autonomy*, we turn to the material, economic, cultural, and informational prerequisites that comprise the sociotechnical basis of autonomy. We end with conclusions (*Conclusion*).

## Aspects of Autonomy: *Capacities, Requirements, Respect, Self-Respect, Exercise,* and *Resources*

This section outlines a multi-dimensional model of human autonomy, providing multiple lenses through which one can analyze the effects that AI systems may have on human autonomy. We make an uncontroversial assumption that human autonomy is at least possible in earlier phases of history without AI technology and that most of what we know of human autonomy is relatively independent of AI. Some theorists propose a relatively minimal [or “a lightweight, ecumenical” (Rubel et al., 2021)] conception of autonomy to maximize compatibility with rival views. We hope to put forward a maximally informative account of autonomy, which is hopefully helpful even for those, who upon consideration do not accept all of the aspects listed here as genuine aspects of autonomy. This is a revisable model aiming to be relatively comprehensive.

The model outlined here contains the following dimensions of autonomy: 1) potential and developed capacities for self-determination; 2) the normative requirements or duties of others (and oneself) to respect and support one’s autonomy; 3) the recognition or respect by others, as a response to such requirements, and the relational autonomy this constitutes; 4) self-respect and other forms of positive self-relation; and 5) the performative aspect of realization or actual exercise of the capacities. Furthermore, 6) various material, economic, legal, cultural, and informational resources can be understood as comprising necessary preconditions for autonomy in these dimensions (These further conditions and resources will be discussed in more depth in *The Prerequisites and Sociotechnical Bases of Autonomy*, the first five aspects in this section). All of the aforementioned aspects are needed for full autonomy, and none of them alone is the full picture, although sometimes one-sided theories stressing only one of them are put forward. As we will see, AI can be relevant in different ways in these dimensions.

Before we proceed, it is worth noting that human autonomy is here distinguished from a minimal “engineering” sense of *functional autonomy*, which refers to a system’s capacity to operate independently, without external agents’ control. Functional autonomy can be ascribed to animals from bees to buffaloes and to some machines, such as robot vacuum cleaners. *Human* autonomy as we understand it is the more demanding notion of autonomy as self-determination or self-rule: it incorporates functional autonomy alongside an adequate degree of control over one’s instincts and impulses, which most animals lack. It is a form of personal autonomy which humans enjoy, at least potentially and to varying degrees, and one essential to the concept of (moral) responsibility (see, e.g., Ripstein, 1999). It is tied to the practical rationality that humans are capable of: the capacity to assess reasons for action and to pursue things that are taken to be of value and the capacity to say “no” to irrational impulses. We do not here take a stand between rival theories of practical rationality, but note that, practical rationality is intimately tied to personal autonomy. While many animals and intelligent machines may engage in instrumental reasoning, they are not capable of weighing the value of novel ends but are provided their fundamental ends by instincts or programmers. Furthermore, we do not focus on collective, moral, or political autonomy, or the mere (fallible) *sense* of autonomy (cf. Pirhonen et al., 2020) as it is a distinct property from personal autonomy itself, although promoting individuals’ sense of autonomy constitutes a desideratum for AI systems’ design and use as well (and, as we note below, self-respect includes a sense of oneself as an autonomous being and is arguably a constitutive aspect of full autonomy). Accordingly, we do not discuss empirical research on *perceptions* of autonomy in the context of AI. Our focus is primarily philosophical, theoretical, and conceptual. The proposed model can however serve as a conceptual foundation for future empirical research, including people’s experiences of autonomy.

### Capacities and Requirements

Self-rule, self-governance, or self-determination (*autos*, “self”, and *nomos*, “rule”) is the essence of autonomy: “The ability to self-govern includes the ability to develop one’s own conception of value and sense of what matters, to [develop] the values that will guide one’s actions and decisions, and to make important decisions about one’s life according to those values where one sees fit” (Rubel et al., 2020, 550). Personal autonomy in humans is commonly understood as this type of agential self-governance or self-determination. In principle, autonomy is a relevant adverb (“autonomously”) for the full range of human thought and action. Objects of self-rule include beliefs, deliberative processes, and other governing principles of action, such as values and desires, as well as particular actions, such as choosing among alternatives or consenting to the interference or guidance by others. Accordingly, autonomy covers *cognitive* and *practical* aspects.

Why should autonomy be respected or promoted? The short answer is that simply because autonomy as self-rule is a valuable thing, and all valuable things are to be respected or promoted and autonomy is no exception to this rule (Raz 2001). It can be added that autonomy is important not only in itself but also as one of the most important constituents of well-being, at least according to the so-called “self-determination theory” or SDT (Ryan and Deci, 2017), and as an important, but not sole constituent of human dignity (Riley and Bos, 2021). Autonomy is to be respected because autonomy is valuable in itself and valuable as a constituent of well-being and human dignity.

Whose autonomy is worthy of respect? This may seem to be a confusing question—surely everyone’s who is autonomous, right? At first hand, the answer “everyone who as a matter of fact is autonomous has the right that their autonomy is respected and protected” may seem appropriate, but it turns out to have a moral loophole: suppose some people (due to discriminatory upbringing, say) are not allowed to become autonomous and so, in fact, do not lead an autonomous life. If only those who “as a matter of fact are autonomous” have the right to have their autonomy respected, then we do not have a moral basis to criticize such discrimination as a violation of autonomy (the violation is that the victims are not allowed to be or become autonomous). The remedy is to appeal to *capacities and potentials*, even hindered ones, as the grounds of the normative demands (Laitinen, 2007). Capacities are the first aspect of autonomy in our model.

So, the moral requirements of respecting and recognizing autonomy are 1) grounded on capacities: the human capacities for self-determination enable a qualitatively different, valuable, form of living, where one forms one’s own valuations and acts accordingly. The capacities ground the right for self-determination and the duties of others to respect it (and arguably even the duty to self to respect oneself as an agent capable of self-determination). Social recognition and interaction are important mediators fostering the development and realization of human capacities. Normal humans are born with the *potential capacity* for self-determination, in which upbringing and exercise mature into a *developed* “*full*” *capacity* at some point between infancy and adulthood. The “fullness” of the capacity is defined as a “range property” (Rawls, 1971), i.e., sufficient capacity for self-determination. Agents with developed capacities have (unlike children) the full right to self-determination and are fit to be held fully responsible for their deeds. If one has a lower capacity than that (due to one’s age or disability), one has a right to *take part* in the autonomous governance of one’s life. The capacities for self-determination are interpersonally and contextually malleable (see Mackenzie and Stoljar, 2000). The developmental stage of growing to be autonomous is clearly a special phase in life, where effects of AI or digitalized environments raise special concerns (Unicef, 2021).

The mis-development of the innate potentials so that one does not develop a full capacity for full self-determination may be caused by various factors. For example, one may lack cultural models or social support for it, one may be actively prevented from becoming autonomous (e.g., think of oppressive structures and discrimination based on gender, “race”, or caste)^1^, or one may suffer from an interiorized sense of inferiority and lack the requisite self-respect.

What kinds of normative requirements do the capacities for autonomy generate? It is widely agreed that the requirements are stringent duties, not merely optional reasons for action that one may or may not adopt. Only more stringent duties may override the duty to respect everyone’s autonomy: they are weighty *prima facie* duties that are overridable but only by more weighty considerations. So, the second aspect in our model is duties and corresponding rights. 2) The normative requirements that autonomy gives rise to are duties and corresponding rights. It is wrong to treat someone as incapable of self-governance if the capacities are there; it is wrong to treat someone as not having the potential to develop the capacities if the potentials are there. Proper respect or recognition of autonomy is a *prima facie* moral *obligation* that everyone universally faces concerning everyone else: autonomy is an important aspect of universal human dignity and creates universal demands. (In *Ethics Concerning Non-Agents: Ought-To-Be Norms and Quasi-Respect*, below, we will discuss the sense in which AI systems can be said to face or “respond” to normative requirements, as they are not moral agents.)

### Respect and Self-Respect

The next two aspects in our model arise from the observation that autonomy has a relational aspect. Respect from others partly constitutes autonomy, and that the same goes for self-relations: it, too, constitutes a further aspect of autonomy.

The importance of 3) interpersonal respect or recognition is closely tied to the fact that humans are born as merely potentially autonomous persons and need recognition and respect to develop their capacity for self-determination. It is clearly wrong and discriminatory to systematically block some people (due to their gender, “race”, or caste) from developing their capacity for a self-determined life. When others respond by recognizing the person as autonomous and respecting them, a *relational* aspect of autonomy is formed. This can take the form of mere “thin” respect from a distance (not preventing them from living autonomously), or it can be a matter of “thicker” interaction (which supports the development to be autonomous). On theories of relational autonomy, this social aspect is directly constitutive of autonomy, not merely a precondition of its development of exercise (Honneth, 1995; Mackenzie and Stoljar, 2000; Kauppinen, 2011). Typically, it is seen as one aspect of autonomy, alongside capacities and exercise thereof—social recognition isn’t everything there is to autonomy. The relevant contrast cases are lack of recognition and misrecognition (disrespect).

One distinction in the relational or social aspect of autonomy is that between directly respecting and indirectly promoting autonomy. In algorithmic and digitally governed contexts, respect (or quasi-respect) for human autonomy can be a matter of the intrinsic functioning or specific functionalities of sociotechnical systems, whereas the indirect promotion or hindering concerns the unintended effects of the widespread use of technologies. The former matters relate to, for example, the meaningfulness of consent, to alternatives available to individuals, to the information provided to them, and also to the control individuals have over their data, and the way it is used. The latter includes ways in which algorithmic technologies and sociotechnical practices, more broadly, affect people’s (capacities for) practical personal autonomy by affecting what alternatives for living one’s life are available, for example. The extent to which technological mediation in broader social, material, and political relations promotes or poses obstacles to individuals’ self-determination is, of course, integral to how collective, democratic, and moral autonomy take shape, respectively. (And as mentioned, we will discuss below in *Ethics Concerning Non-Agents: Ought-To-Be Norms and Quasi-Respect* whether AI systems can directly recognize or respect human autonomy, or whether it is better to talk of quasi-respect.) Another important distinction is that between interpersonal (informal, horizontal) and institutional (formal, legal, vertical) recognition of autonomy. In principle, all forms of respect can take an institutional form, most importantly, that of legal recognition.

Corresponding to social respect, autonomy requires 4) self-respect from the agents. It is a form of relation-to-self that is arguably constitutive of the state of being autonomous. Such self-relations are greatly supported by recognition from others (Honneth, 1995). The operative conception of others (“you are not capable of self-determination, because you are an x”) can be internalized into a crippling self-image (“I am not capable of self-determination, because I am an x”). Arguably, self-respect is, analogously to social respect, a response to the normative demands based on having the capacities. An agent wrongs oneself fails to meet the duties to self, in not treating oneself as an equal person among others, or in not being sensitive to the full relevance of the capacities one possesses.

### Exercise of Autonomy and External Resources

The final two aspects in our model relate to the sense in which full autonomy is realized in action that the capacities and relations enable. But further, external resources are needed in such exercise. Effective 6) *exercise* of autonomy in relevant (autonomy-constituting) activities counts as its actualization or realization: leading an autonomous life consists of actual actions, decisions, human conduct, and thinking. In the multidimensional model that we propose, the performative aspect of autonomy is also constitutive: there is a constitutive aspect missing from one’s autonomy if one has the developed capacity for self-determined life but does not exercise it—even when that capacity is duly recognized by others. This is the case when one lives *heteronomously*, which is the contrast case of autonomous life. Instead of determining one’s views and actions by oneself, one is governed by something other than oneself. In Immanuel Kant's (1996a) view, the ways of being heteronomous include blind obedience to tradition and authority, without forming an independent view of one’s own, and, of course, downright manipulation and coercion (which are cases of interpersonal disrespect at the same time). But interestingly, being a “slave of passions”, and acting on one’s desires, whims, and inclinations without caring whether one ought to act on them, is for Kant (Kant (1996b)) a form of heteronomy as well. So is a mere arbitrary choice without considered reasons for one’s actions or thoughts. So interestingly, the real self is the *rational* part of oneself, whereas blind will, uninformed existential choices, or bodily or mental urges do not constitute the real self.

In any of these dimensions, a spectrum of cases from very weighty or important can be considered, and violations of autonomy are a very grave matter when weighty matters are at stake, whereas in more trivial cases, the violations matter less. In Charles Taylor’s (1985b) example, hindering religious freedoms is an important matter, whereas having to stop at red lights is not really a violation of one’s autonomy at all (although in some sense interferes with one’s activities). There are degrees of importance: in judging what supports or violates autonomy, we are implicitly relying on a normative understanding of the importance of autonomy. It is an ethical, evaluative, normative concept and not merely descriptive or evaluatively neutral, or impartial one. In judging whether traffic lights can constitute a violation of autonomy, we draw on an understanding of what is important in human life. This suggests that even the liberal ideals of neutrality or impartiality, which typically demand universal respect for autonomy, and which may be important aims in diverse societies, are also value-laden (and in that sense non-neutral, or what philosophers call “perfectionist”) principles, drawing on particular evaluative stances and particular understandings of autonomy (Taylor 1985b; Raz 1986). If this is indeed the case, demands for supposedly neutral respect for autonomy cannot but draw on particular understandings of autonomy. Rival understandings of autonomy lead to rival views to what universal respect for autonomy amounts to. The more multifaceted and multidimensional the conception of autonomy, the better it is as an evaluative starting-point, and the less likely it is to miss important aspects of autonomy.

So far, we can collect these aspects in a Table 1. How could it come to be, that one does not exercise autonomy even when one has the opportunity? To see this, it is good to note that genuine autonomy is more than negative freedom. Obstacles to negative freedom are obstacles to autonomy: one can lack autonomy and negative freedom by lacking choice between alternatives, or especially by lacking *meaningful* alternatives, or resources to pursue them. Or, one may have the choices made by others. In the cases of adults, even well-meaning *paternalism* is a violation of self-determination, because the adults have the developed capacity for autonomy.^2^ But in some cases, one may exercise one’s negative freedom yet not exercise one’s autonomy. Choosing among alternatives in an arbitrary manner, without reasons, by blind deference to authority, or on a whim, would constitute such cases. At issue, here is not the absence of (meaningful) options but, rather, the authenticity or rationality of one’s deliberative processes. These cases can be called “heteronomous”, and they may suggest that the agent herself lacks self-respect, perhaps, due to an internalized inferior self-image. Autonomy in the fullest sense entails that individuals choose (to act) freely on the basis of reasons they can understand and endorse, without (a history of) manipulation. This is the sense in which we talk about *autonomous will*. Importantly, this type of autonomy does not require full coherence over time in deliberation or action; autonomous does not imply full conformity to one’s past choices. Decisions and judgments, while more stable than whims in remaining unless revised, are revisable (but autonomous revisions should be distinguished from arbitrary choices).

**TABLE 1:** Dimensions of human autonomy.

*The dimension of personal (cognitive and practical) autonomy*	*The contrast case*
Capacities	Lack of potentials or of their development
Requirements	(merely apparent requirements; e.g., moral nihilism or error-theory)
Respect from others	Various forms of disrespect, manipulation, coercion, etc.;
Self-respect (and the other psychological conditions)	Lowered self-respect (and lack of the other psychological conditions)
External resources (material, economic, cultural, informational)	Insufficient resources
Actual exercise, autonomous life	Heteronomous life despite real opportunities

Lastly, it should be noted that exercise of autonomy further requires various 5) external resources which can be understood as necessary conditions for the aspects of autonomy considered above. Such resources range from material and economic resources to cultural and informational prerequisites. We will take a closer look at these in *The Prerequisites and Sociotechnical Bases of Autonomy*, where we briefly consider the relevance of algorithmic systems for the existence and distribution of such resources and prerequisites. These resources are external in the sense that they do not themselves *constitute* autonomy but maybe its necessary prerequisites: humans need, for example, oxygen to live, and they need to live to be autonomous, but access to oxygen is not as such yet any aspect of autonomy, merely its precondition. Such external preconditions vary from material, economic, and legal to cultural and informational resources.

## Ethics Concerning Non-Agents: Ought-To-Be Norms and Quasi-Respect

We have seen that human capacities for autonomy ground duties that others have: they ought to respect human autonomy. What does this imply for AI systems? Before a more detailed discussion on the normative requirements that protecting or respecting human autonomy poses for other human agents and AI systems, we need to address a worry: does it even make sense that AI systems should respect human autonomy if they are “mere machines” without a conscience?

Physical nature can be an obstacle to human autonomy, but nature does not face any normative demands. Human agents can be obstacles to each other’s autonomy, and face duties and other normative requirements to act. The principles govern what they ought to do and are thereby “ought-to-do” norms. In this section, we argue that AI systems belong, with other artifacts, to an interestingly different category: they are not moral agents and do not literally have duties. Yet such artifacts are not entirely free from normativity, like stones or rivers whose movements can be explained by laws of gravity and natural forces. Our suggestion on how to apply the interhuman norm of “respect for autonomy” to human-AI relations is as follows: Even though machines are not capable of recognition or respect—they do not belong to the class of recognizers—and thereby do not have duties and ought-to-do norms do not apply to them, there are so-called “ought-to-be” norms that apply to them.^3^ Let us explain.

Only moral agents can have duties to act in certain ways. It is not *a priori* impossible that some artifacts could in the future meet the conditions of moral agency even though none seem to meet them at the moment. Experts disagree on whether any robots or AI systems are, and whether they should be, moral agents now or in the future (cf. Gunkel 2018; Himma 2009; Coeckelbergh 2009). To the extent that a technological artifact is indeed a moral agent, then it has duties to respect human autonomy. What everyone can agree on is that there are some technological systems that are not moral agents, but which nonetheless can be obstacles to human autonomy. Some of them perhaps *simulate* moral agency, but what matters here is whether or not they are moral agents (Hakli and Seibt 2017). An interesting philosophical question concerns those artifacts that are not moral agents and cannot bear duties, but which nonetheless can harm humans and worsen their prospects of autonomous action. Can there be any normative requirements concerning such systems?

Such artifacts, even though they function or even “act” in certain ways (and can be said to “treat” different classes of humans and animals in certain ways, for example) which are not fit to be held responsible and which do not meet the conditions of moral agency, do not have duties. This article (putting aside for now possible future artificial moral agents) explores the idea that instead of having literal duties, there can nonetheless be so-called “*ought-to-be norms”* concerning such nonmoral or non-responsible artifacts. (On ought-to-be norms, see Sellars, 1968; Wedgwood, 2007; Tuomela, 2013). To illustrate the idea of “ought-to-be” consider naturally evolved organs such as hearts, or artifacts such as clocks, and the phenomenon of not functioning or being broken: hearts ought to be such that they pump blood, but they do not have *a duty* to pump blood. It is just what their function is, what they ought to be like, or otherwise, they are dysfunctional, impaired, broken. Similarly, clocks ought to showtime. This sounds like an ought-to-do norm (if “showing time” was something they ought to do in order to fulfill their duty), but in fact, it is an ought-to-be norm: clocks ought to be such that the passage of time can be read from them, on pain of being broken or dysfunctional.

What we suggest here is that AI systems are in this respect like hearts or clocks, and in addition to being designed for specific tasks, for them to be ethically acceptable; they *ought to be* such that they (among other things) are not obstacles to human autonomy. A further idea is that the content for those ought-to-be norms can be derived in a victim-based way from how they affect the affected parties, how they “treat” the patients, victims, or recipients. Once we understand what the duties for moral agents would be, we can understand what machines (with a sufficiently similar range of functional abilities) ought to be like. For example, whereas moral agents, governed by ought-to-do norms, have negative and positive duties to promote and respect human autonomy, concerning nonmoral artifacts, there are ought-to-be norms to “promote” and “respect” human autonomy. Machines (especially intelligent machines) *ought to be such that* they do not prevent human autonomy. Violations of autonomy are the content that in the case of agents, creates *duties*, but in the case of machines, creates *ought-to-be norms* with the same content.^4^

It is helpful to keep in mind that moral predicates (permissible, impermissible) concern all alternative courses of action open for a moral agent. Similarly, a machine in a situation has alternative functionings, and all of them are either “OK” or “not OK” in light of the ought-to-be norms concerning that machine. The repertoire of functionings will typically be different for a machine (with different shape, size, and number of arms, etc.) and for human agents, but nonetheless, all the functionings available (everything that the machine can “do”) are normatively evaluable as acceptable or not. Even if the repertoire of functionings by a machine that causes risks for human health is different from the repertoire of human actions that cause risks, nonetheless, the risk for human health can make the actions or functionings “not OK”. The same goes for risks to human autonomy.

Are there differences between what humans are permitted to do, and what AI systems are permitted to do? To use the well-worn-out example in a new way, is what a human trolley driver or bystander is morally permitted to do in a runaway trolley scenario the same as how an automated trolley and switch–system ought to function in a similar scenario? And is what a non-responsible automated trolley and switch–system ought to “do” in a scenario the same as what a responsible (i.e., one meeting the conditions of being fit to be held responsible) automated trolley and switch–system, should there someday be one, ought to literally do in the same scenario? Other things being equal, the demands created by the autonomy, dignity, *etc.* of the potential victims apply to both humans and AI systems. They both are to be, in their own ways and repertoires, responsive to the values of human autonomy and dignity. Yet, other things need not be equal: one difference is that human agents have prudential reasons, and they need not sacrifice themselves for other humans, but presumably, machines do not have such rights to self-preservation. (We thank an anonymous referee for stressing the importance of this).

The approach adopted here has two important features: Firstly, it is a victim-based (or patient-based) approach to the content of moral duties and ought-to-be norms: effects on the human autonomy (and dignity, well-being, justice, etc.) of the victims are the entry point to assessing what moral duties agents have and what machines ought to be like. There are, of course, various ought-to-be norms deriving from the function or purpose of the machine itself, so the victim-based approach concerns (quasi-deontological) side constraints and (quasi-moral) limits of their functionings. Whatever their main aim is that aim ought not to be pursued in ways detrimental to human autonomy. Secondly, it can be called a simulation approach in that it first asks what duties moral agents would have in a situation and then asks what machines ought to be like in similar respect *as if* the machines were moral agents.

We can call breaches of the negative duties *violations* of human autonomy and call a successful meeting of the negative duties *respecting* human autonomy. Furthermore, we can call breaches of the positive duties *neglect* of human autonomy and call a successful meeting of the positive duties, typically by engaging in right kinds of activity, *positively supporting* human autonomy.

What relevance do these distinctions have for considering the role of AI in securing human autonomy in today’s sociotechnological forms of life? To answer this, the simulation approach suggests it is helpful to start from the duties that *moral agents* have. If robots or AI systems are moral agents, they have such duties literally. If they are not moral agents, they should arguably nonetheless be built to be such agents so that they function accordingly; they *ought to be* such that the autonomy of moral patients is not violated but is supported. Concerning any artifacts, there can be such ought-to-be norms literally (just like hearts and clocks ought to be such that they pump blood or show time), even if they would not have ought-to-do duties; clocks ought to be such that they show time reliably, chairs ought to be such that they do not collapse under the human weight, and so on. Many kinds of responsibilities and ought-to-do norms follow from these for agents; clockmakers ought to make clocks that work, salespeople should warn customers if the clocks they are selling are not very reliable, and people agreeing to meet each other should warn each other if their clocks are not reliable in keeping time. Concerning robots and AI systems, such responsibilities are, or should be, similarly distributed to engineers, salespeople, users, maintenance people, and legislators.

To recap, only moral agents literally have duties. Nevertheless, robots ought to be built so that they do not harm yet protect vulnerable humans and help meet human needs. They *ought to be* such so that they do not block people’s autonomous agency, rational thinking, or equality but rather are of help in aiding and supporting those aims. Thus, the same list of concerns can be construed as a list of ought-to-be norms based on human autonomy, applicable to robots and AI systems even when they are not moral agents themselves. The responsible designers, users, etc. of AI systems then have ought-to-do norms which correspond to these ought-to-be norms: they ought to see it that the AI systems are of the appropriate kinds. Let us next turn to take a closer look at the content of the demands that human autonomy poses to moral agents and AI systems, with examples drawn from the literature.

## Interpersonal Disrespect and Violations by AI Systems

There are several kinds of obstacles for human autonomy which undermine or restrict the development or exercise of individuals’ capacities for self-determination (Table 2). These obstacles are relevant to AI and its effects insofar as they affect individuals’ capacity for, or exercise of, autonomous self-determination.

**TABLE 2:** Forms of interpersonal disrespect and their corresponding ought-to-be norms for AI systems.

Form	Description	“Ought-to-do” norm (*Prima facie*)	“Ought-to-be” norm (*Prima facie*)
Direct interference	A physically prevents B from doing X	A ought to respect B’s freedom to do X	System A* ought not compromise B’s freedom to do X
Coercion, threats, naked power	A forcing B to (choose to) do X instead of Y	The human agent A ought not coerce B	System A* ought not quasi-coerce B
Manipulation, indoctrination, deception	A manipulating B to value and desire X (or X instead of Y), creating in B a disposition to X	The human agent A ought not manipulate, indoctrinate or deceive B into doing X (instead of Y)	System A* ought not manipulate, indoctrinate or deceive B into doing X (instead of Y)
Nudging	With environmental cues, A goading B to do X rather than Y (when B has a predisposition to do either)	Any intentional priming should be such that it can, when asked, be openly declared, known, and accepted	Any quasi-intentional priming should be such that it can, when asked, be openly declared, known, and accepted
Paternalism	A deciding on behalf of B, benevolently guided by A’s judgement about what is best for B	The human agent should not interfere with B’s decision regarding what is best for B	System A* ought to be such that it does not interfere with B’s decision regarding what is best for B
Cognitive heteronomy	B willingly defers to A instead of forming one’s own judgement	Other agents’ autonomy and positive relations to self should be supported, and heteronomy discouraged	System A* should support human agents’ autonomy and positive relations to self, and discourage deference
Direct misrecognition, denial of autonomy	A not regarding B as capable of, or possessing the right to, self-determination	The human agent A should not fail to recognize B’s (capacity for or right to) autonomous agency	System A* should not “send a message” that B is not capable of, or possess the right to, self-determination
Misrecognition, denial of preferred labels	A not regarding B in light of the particular self-understandings that B has autonomously self-defined	The human agent A should regard B in light of the particular self-understandings that B has autonomously self-defined	System A* should allow for B to be regarded in light of the particular self-understandings they have autonomously self-defined

We will now discuss ways in which AI can be an obstacle or support human autonomy. Given that interpersonal disrespect can take various forms and that the nature of many specific violations of human autonomy, such as manipulation, can vary depending on the use-context and task executed by the AI system, the following overview is non-exhaustive. Nonetheless, we believe it covers most ethical discussions and debates found in the literature on AI and human autonomy. Furthermore, the aim of this section is to clarify these debates by pointing out some misconceptions about the normative requirements human autonomy poses for the design of AI systems. We hope that our multidimensional model helps to analyze AI systems in terms of their effects on autonomy at different levels of technology experience (for similar analyses, see Calvo et al., 2020).^5^

### Direct Interference

AI systems can obstruct human practical agency by limiting a person’s negative freedom. Relevant examples are neither hard to find nor to imagine: AI systems are increasingly integrated into equipment and vehicles that can pose risks for physical harm, delivery robots can obstruct pedestrians’ movement, facial recognition software used for unlocking smartphones can fail to recognize their users’ faces, and so on. The relevant negative freedoms vary significantly depending on the technology and use-context. Technologies that can exert relevant influence on the physical environment can interfere with individuals’ physical functions and mobility, while others, such as the facial ID software, can prevent access to goods and resources and hinder human connections. The scope of possible risks here depends on a given system’s use-context, its physical “embeddedness”, and its capacity to affect the physical environment of operations but also on the degree of accuracy, robustness, and consistency such systems exhibit in their operations. When AI systems perform well in the latter respects, they can plausibly also prevent physical harm, and even support individuals’ physical functioning and mobility.

### Coercion, Manipulation, and Deception

Coercion consists in the removal of meaningful options, or offering options one cannot refuse, without interfering with one’s reasoning about those options (see Susser et al., 2019). Plausibly, most extant applications, such as recommender systems, are not coercive in this sense. When Spotify recommends its users songs, it does not coerce the user to play those songs. In comparison to orders, or cases of downright forcing, such recommendations are sensitive to users’ autonomy. In general, recommendations and personalized choice architectures can be of assistance and provide food for thought. Of course, users may lack *meaningful* alternatives in a broader sense—say, one’s preferred niché genre of music may be not represented in Spotify’s catalogue of songs. Such cases can be understood as involving not a direct disrespect for autonomy but rather a lack of diversity in cultural resources, which we discuss in *Cultural Resources*.

It is an implausible claim that recommendations by machines would *inherently* undermine autonomy. However, specific *contingent factors* related to the use of AI systems rightfully raise concerns about manipulation and deception. For example, so-called “hypernudging” on various platforms and applications—a dynamic, highly personalized, and often opaque form of regulating individuals’ choice architectures through Big Data techniques—would seem to rightfully raise such concerns. The same goes for issues with transparency and privacy, for example (Yeung, 2017; Lanzing, 2019; on nudging; see Thaler and Sunstein, 2008).

Susser and colleagues have defined *manipulative* algorithmic practices as “applications of information technology that impose hidden influences on users, by targeting and exploiting decision-making vulnerabilities” (2019, 29; italics omitted). As they explain, manipulation differs from mere persuasion; although they both work towards the similar aim of having one agent work towards the other’s goal, persuasion uses rational arguments and incentives as its means, while manipulation uses hidden influence. Likewise, manipulation differs from coercion in that the manipulator interferes with the subject’s reasoning as opposed to (merely) the option space. Deceptive technologies can be manipulative when they instill false beliefs and thereby interfere with how the human individual’s reason, to further the manipulator’s aims. But all manipulation is not deceptive, and all deception is not manipulation. For example, according to Susser and colleagues, the well-known Cambridge Analytica case of targeted political advertisement constituted manipulation without deception (Susser et al., 2019). Some extant self-tracking health technologies could be understood as manipulative as well: while they are envisioned to promote users’ autonomy by making themselves transparent through quantification of their behavior, these applications employ non-explicit psychological strategies to bypass users’ autonomous will and incentivize comparison to others through “co-veillance” mechanisms, making one’s choices possibly inauthentic (see Lanzing, 2016). There are also deceptive AI technologies, such as “deepfake” generators, which can be used for manipulative purposes as well as for simple entertainment.

Concerning recommendation systems, the relevant worries regarding the effects that hypernudging has on persons’ cognitive and practical agency plausibly relate to the *degree* and *scope* of nudging, not its modality or kind (see Danaher, 2018). The fact that nudges and recommendations are continuous, personalized (or targeted) and dynamic, does not change their nature as recommendations, although other adjacent harms and issues related to recommendation systems can be exacerbated as a result (e.g., the spread of misinformation). Contingent issues, such as the adjacent lack of transparency and privacy (see Lanzing, 2016; Lanzing, 2019; Susser et al., 2019), may alter the acceptability of recommendation systems. Indeed, hypernudging by AI systems can affect our thinking through *opaque* nudges; such recommendations and influences do not reveal their own functioning and can thereby be autonomy-undermining. However, insofar as recommendations wear their nature as recommendations on their sleeves, they provide options instead of bypassing thought and autonomous choice.

The takeaway here is that recommendation systems and personalization can be autonomy-supporting insofar as they provide meaningful alternatives in a transparent manner. AI systems that recommend, nudge, and personalize can fail to be respectful of human autonomy due to adjacent, contingent factors (e.g., opacity) and can be harmful in other ways due to their scope of influence.

### Nudging and Paternalism

Nudging that seeks only further interests that the nudged person could not reasonably endorse is arguably morally problematic. It is less clear, however, whether and when nudging that falls under so-called “benevolent paternalism” is justified. When (if ever) is one allowed to interfere with others’ autonomy in ways that are beneficial to them? Say, when should recommender systems “nudge” users into making *good* decisions—e.g., ones that align with users’ values? There is no simple answer, but arguably justification for benevolent paternalism requires that at least the following four conditions are met (see *The Prerequisites and Sociotechnical Bases of Autonomy* in Beauchamp, 2019):

(*The Harm Condition*) Were one not to interfere, an individual would face a substantial and preventable harm (or loss of benefit).
(*The Likelihood Condition*) Interference is highly likely to prevent the harm (or loss of benefit).
(*The Weight Condition*) The likely benefits due to interference outweigh the interference-related risks or harms; and
(*The Minimal Interference Condition*) The chosen form of interference is the least restrictive one necessary for securing the expected benefit (or for reducing the expected harm).

What this shows is that the justification of paternalistic nudges depends on the expected benefit or harm in question. The stakes are clearly higher when medical AI systems make treatment recommendations than when users receive product recommendations in online stores, for example.

Nudging and paternalism are *prima facie* violations of autonomy, but when benevolent paternalistic nudging is justified, there is an overriding reason (based on expected benefits and harms) to influence a person’s actions through environmental cues. To respect the nudged person’s autonomous standing, those reasons ought to be aligned with their reasonable interests but also, when asked, openly declared to them.

Respecting a person’s autonomous standing also requires recognizing that person as an individual capable of authoring their own life (see Eidelson 2015). This implies that we ought to not treat users as lacking in their capacity to judge sociotechnical practices. This has been less often discussed in the literature. As Danaher rightly points out in his discussion on hypernudging and personal autonomy, an uncritical narrative of helplessness in the face of AI should be avoided:

“In the world as it is currently constituted, we are not slaves to AI assistance; we do have some residual control over the extent to which we make use of this technology. We have no legal or moral compulsion to use it, and we have our own self-judgment about the effect of certain choices on our happiness and fulfillment” (Danaher, 2018, 645).

In other words, at least when it comes to AI assistants and recommender systems, we often have control over whether we adopt (and continue to use) the technology, how we use it, and how we regulate it^6^. Respect for autonomy requires that we acknowledge this (even if we understand autonomy in a relational or situated manner, and when we rightfully criticize technologies and platforms for obstructing autonomous agency through intentional opacity, for example).

In short, while nudges may constitute transgressions against individuals’ autonomy, they may yet serve individuals’ interests of leading a good life in cases where nudges work to further individuals’ long-term goals and aims.

### Cognitive Heteronomy

In *Exercise of Autonomy and External Resources*, it was noted that cases of heteronomy often suggest a lack of self-respect, the presence of manipulation, or other obstacles to autonomous conduct. This issue has been discussed in the literature on AI systems as well. The delegation of cognitive tasks to assistance technologies, such as AI assistants and health applications, has been feared to have degenerative effects in that they would impoverish humans’ cognitive capacities needed for autonomous agency (see Danaher, 2018). There are two questions here, reflecting the distinction between capacities and exercises: are we 1) deferring to the judgments of others in a non-autonomous manner, or 2) impoverishing our capacity to author our lives?

Danaher (2018) notes (correctly) that there is no simple answer. Delegation of certain tasks to AI is arguably not a significant threat to the exercise of autonomy if it is uncoerced and not a result of manipulation. Insofar as delegation is deliberate, consensual, and opens up new possibilities for autonomous action (e.g., time for more meaningful tasks), it does not compromise autonomy in itself. The decision to delegate a given task can be an autonomous act. Furthermore, recalling Taylor’s (1985b) suggestion, there are also degrees of importance in what we consider as essential for autonomy (see *Exercise of Autonomy and External Resources* above). Certain practices are essential to how we conceive of ourselves as persons, others less so. Furthermore, the AI assistant may operate (more or less) in accordance with a user’s values and interests—giving the user (stronger or weaker) reasons to endorse the recommendations.

The second question is whether AI technology takes us too far in this respect, having a degenerative effect on our *capacity* to live autonomously, e.g., whether our capacity to be mindful of our values and goals (regarding, e.g., well-being) will become impoverished, or whether the habit of actually executing the given task (as opposed to delegating it to technology) is essential to maintaining our physical and cognitive capacities. As Danaher argues, determining whether a given type of use of an AI assistant will have degenerating effects that are damaging and widespread will depend on the role of that task in the individual’s life, and “the possible need for cognitive resiliency with respect to that task” (Danaher, 2018, 639). One needs to assess whether the possible degenerative effect on capacities is non-localized, compounding on multiple areas of one’s life, and whether the net effects on autonomy as a result of the delegation are positive. Such questions are answered by assessing AI systems’ effects on different levels of technology experience and by looking at “the specific ecological context in which AI gets used and the impact it has on cognitive ability, freedom and responsibility in those contexts” (Danaher, 2018, 646).

### Direct Misrecognition

As stated in *Respect and Self-Respect*, one blatant form of disrespect for autonomy is the active denial of one’s autonomous standing, or a failure to recognize it (see Honneth, 1995; Eidelson, 2015). There are at least two specific ways recommender systems and automated decision systems could “by default” fail to be sensitive to autonomy in these senses.

Firstly, recommendations and predictions based on the past, on individuals’ historical data will remain imperfect because leading an autonomous life may involve changes in one’s habits, preferences, and character. As recommender systems typically track only individuals’ first-order preferences or desires by surveilling their actions at the level of data (e.g., clicks), they can remain insensitive to changes in users’ preferences about their desires and preferences (Frankfurt, 1971). For example, a cigarette smoker may want a cigarette but may want to stop wanting cigarettes. Individuals may want to change their consumption habits or long-term goals. That AI systems typically cater to individuals’ first-order preferences can be particularly detrimental to autonomy when first-order preferences reflect individuals’ *addictions* or *akratic will*—both hindrances to the cognitive and practical agency. Think, for example, of a gaming addict being recommended the latest online games (perhaps, despite their conscious effort to fight the addiction). In principle, recommenders can try to take such second-order preferences into account: there may be interface options for opting out from previous patterns, and other kinds of ongoing tailoring of recommendations (e.g., “do not show me this kind of content”). The presence of options would increase sensitivity to changes in individuals’ values, preferences, and second-order preferences.

Secondly, some have raised concerns that the intrinsic functioning of data-driven systems fails to respect the autonomous standing of persons. Given that recommenders and decision systems “regard” an individual always with reference to others—i.e., they “treat” them as mere members of a group and not *as* individuals—it seems they would fail to respect the individuality of each person. For example, the recommendations individuals receive are typically collaboratively filtered and thus always involve (at least implicit) reference to others. This issue concerns any system that bases decisions on group-level statistics. Indeed, some have argued that “[b]eing subjected to algorithmic decision-making threatens individuals’ personhood by objectifying them” by default, and this can consequently “defeat [their] autonomy” (Kaminski, 2018, 1541). Empirical research also supports the notion that algorithmic decisions involve risks of objectification, of individuals being reduced to mere numbers or percentages, as it were (Binns et al., 2018).

If decisions based on group-level, statistical probabilities were to undermine human autonomy, then, AI systems would prove intrinsically worrisome in this regard. This worry seems misguided, however. Generalization is not only unavoidable in practice, and in many cases, it is all-things-considered morally acceptable and, in some cases, perhaps even morally required (Lippert-Rasmussen, 2011). More importantly, generalizing and treating people as individuals are compatible so long as the information that decision-makers rely on captures morally salient facts about persons with a sufficient degree of granularity, whether that information is statistical, non-statistical, or both (Lippert-Rasmussen, 2011). In other words, the degree to which we can hold an AI system to treat us as individuals is dependent on whether the informational bases for treatment are sufficiently tuned to the normative requirements of decision-making contexts—e.g., whether reasonable individuals would endorse the use of certain information (regardless of its “type”).^7^ There may yet be other moral or political reasons to refrain from decision-making based on past data or probabilities—in a democratic society, for example, everyone ought to be given a right to vote regardless of how they’ve used their vote previously or how they intend to use their vote in the future.

In sum, various technologies, such as AI assistants and recommendation systems, can promote individuals’ autonomy by offering them alternatives tailored according to their needs and by helping them to conduct meaningful tasks. Violations of autonomy in the context of AI are often contingent on various factors. Malfunctions or bad performance can lead to undue direct interference or violations of negative freedoms; recommendations can transpire into manipulation unless transparent; nudging can be autonomy-violating when unaligned with the nudges’ values and meta-preferences, even when benevolently paternalistic in that it serves individuals’ best interests. Floridi et al. (2018) rightly suggest that in most cases, it seems especially important that people “retain the power to *decide which decisions to take*, exercising the freedom to choose where necessary”. This kind of “meta-autonomy”, as they call it requires that necessary mechanisms for consent and transparency are in place.

## The Prerequisites and Sociotechnical Bases of Autonomy

AI systems may also affect the circumstances and prerequisites of autonomy. By prerequisites, we refer to resources, opportunities, and other things which are themselves not constituent parts of autonomy, but which facilitate it, or comprise its necessary conditions. Broadly speaking, such prerequisites (and corresponding obstacles) range from biological, material, and psychological to social, political, and economic resources and affordances. All these aspects are increasingly technologically mediated and partly interdependent sociotechnical bases of autonomy (see also Hoffmann, 2020). As there are more and less appropriate ways of organizing sociotechnical systems from the point of view of human autonomy, the existence and distribution of these prerequisites may be normatively governed by ought-to-be norms that derive from the value of human autonomy. Our societies and their sociotechnical arrangements ought to be such that they enable humans to live autonomous lives.

### Material and Economic Resources

Prerequisites of autonomy include material and economic resources. In debates on positive freedom, it is often pointed out that *real* freedom, *real* opportunity to exercise one’s autonomy will require material and, in financialized societies, economic resources. Quite simply, one is not in a position to decide whether to eat a particular food or read certain books if one cannot afford them (Van Parijs, 1995). This perspective to the material prerequisites of autonomy requires a focus on how AI systems structure opportunities, allocate resources, and mediate practices across sectors such as education, finance, employment, social security, health care, and whether access to relevant material and economic resources is promoted or obstructed through such mediation. (O’Neil, 2016; Eubanks, 2018).

### Cultural Resources

Culture and cultural resources are equally important in the exercise and development of the capacity to self-determination: if “autonomy” is a relatively recent idea in human history (only some millennia or centuries old), there must have existed human forms of life whose members did not aim at autonomous life because they did not have the very idea available for them (Taylor, 1985a). In addition to the cultural idea of autonomy being a prerequisite for the development of the capacities of self-determination, the availability of a sufficient range of meaningful cultural practices is necessary for the exercise of autonomy. One cannot choose between, say, aiming to become an opera singer or a footballer if those practices do not exist. Arguably, the range of options need not be maximal, but sufficient for autonomous choice to be possible: perhaps, a wider range than merely two options provides a better condition for autonomy than merely two options, but at some point, there is a sufficient range, and mere quantitative addition of more alternatives does not add to the (already “full”) possibility of autonomous choice (Raz, 1986).

When AI is being introduced and developed in cultures where autonomy is well-recognized and respected, the question arises of how AI can support or prevent the cultural preconditions of autonomy, for example, the presence of meaningful options for self-authored lives. One salient question is whether individuals wishing to live lives without technology (for ecological reasons, perhaps) have the genuine possibility to do so. This question aside, it seems AI can have both positive and negative effects with respect to the cultural practices. On the one hand, AI applications in cultural and creative sectors can support cultural practices by improving their accessibility (see Caramiaux, 2020) and by helping create cultural products, such as “AI art”. Algorithmically governed digital platforms, such as those currently owned by Facebook and Google, also offer novel means for “content creation”, for example. On the other hand, one could ask whether the centralization of such channels will narrow down the range of meaningful practices or negatively affect their quality due to operative algorithmic logics of optimization and the capitalist logic of content monetization, for example.

### Psychological and Informational Prerequisites

Various psychological prerequisites to autonomy partly constitute the relevant capacities and relations to self. These range from a sufficient level of understanding (e.g., understanding the relevant options; see the informational resources below) to sufficient independence from urges and inclination and not being compulsive, addictive, or akratic (e.g., not being addicted to digital gadgets), to having the sufficient courage to act in one’s natural and social environment (closely related to positive self-relations such as self-respect). We can think of these as included in the capacities to self-determination, on the one hand, and in the positive relations to self, on the other hand, and we can merely point out that these are relevant as direct constituents of an aspect of autonomy and at the same time prerequisites for the exercise of autonomy. Conversely, insufficiencies and obstacles in these respects obstruct the effective exercise of autonomy.

AI and other digital technologies can form obstacles in this regard. For example, in addition to social, economic, and political structures, “self-respect is also importantly shaped by the design, dissemination, and use of technology” (Hoffmann, 2020). Widely used search engines, for example, can (and do) reinforce discriminatory racial stereotypes and, in doing so, actively shape how users perceive others and themselves (Noble, 2018). Similarly, “deepfake” technology used, for example, to generate “revenge porn” (see Harris, 2018) can lead to experiences of humiliation. In examining how AI technologies *qua* sociotechnical systems can affect the psychological prerequisites of personal autonomy (negatively and positively), one has to take into account different spheres of technology experience, ranging from relevant effects at the level of individuals, groups, and society at large (Peters et al., 2018). Furthermore, it should be acknowledged that different groups may be disproportionately burdened by algorithmic systems that produce demeaning content and generate representational harms that can shape relations of (self-)respect.

Of special interest in the context of AI technology are so-called “*informational prerequisites*” of autonomy. Both transparency and privacy have been discussed as such prerequisites (Rubel et al., 2021; Lanzing, 2016; Lanzing, 2019). Roughly, the former allows an individual to access information required for exercising their cognitive and practical agency according to their self-chosen values and commitments, while the latter safeguards the individual from interference. For example, regarding transparency, Rubel et al. (2021) argue that, especially in high-stakes decision-making contexts, respect for individuals’ autonomy requires (*prima facie*) providing them with information that allows them to act according to their values and commitments (practical agency), and which allows them to evaluate and understand their situation in order to deliberate how to act (cognitive agency). They focus especially on cases where a system hinders human autonomy, but this hindrance can be removed or mitigated by providing the relevant information, for example, about the workings of the system to the affected humans. In such cases, Rubel et al. argue individuals have the right to access data concerning them, and further can make (defeasible) claims to transparency regarding algorithmic systems. While all sorts of sufficient information are relevant for the cognitive and practical agency, Rubel et al. think the normative requirement is clear especially in the mentioned cases, where some system has restricted one’s agency, and getting information would mitigate the effects on one’s agency: one can rationally endorse or object the functionings of a system only once and one knows how it works. Failures to guarantee access to such information can be understood as being on a part with other forms of obstructing agents’ exercise of autonomy, such as deception or manipulation, and insofar, an agent is prevented from meaningfully evaluating their situation (see discussions in Susser et al., 2019; Rubel et al., 2020).

Regarding the converse direction of information flow, Lanzing (2016), Lanzing (2019) has argued for the importance of *informational privacy* (control over one’s personal information) and *decisional privacy* (control over whether and to what extent others may comment, interpret, change, or in other ways interfere with how one leads their life) for autonomy. While the value of privacy cannot, perhaps, be reduced to autonomy considerations, it may be the case that “autonomous decision-making, self-development, or self-presentation [...] cannot be developed or exercised” without privacy, making privacy valuable partly due to its relation to autonomy (Lanzing, 2019, 558). Indeed, having one’s thoughts and actions surveilled and interfered with in ways one could not reasonably expect or endorse in light of their own values and commitments can obstruct one’s cognitive and practical agency. Some information, notably, is “private knowledge”; it is inappropriate for others to even have views about it, and it is a violation of autonomy to “rob” that information from the individual. Similarly, contextual norms of privacy can be violated if individuals’ data starts flowing to directions they would not reasonably expect, for example, when platforms change their data policies on a whim.

Indeed, *informational self-governance* can be considered a specific form of exercising one’s autonomous agency through governing one’s digital representation. Consider social categories based on self-identification (on some theories, e.g., one’s gender). In such cases, others *are* supposed to track one’s self-identification, and they often do so by observing how individuals perform their identity in social contexts, e.g., how they govern their representation of self. If such self-identifications do not receive due recognition, the individuals’ rights to self-definition are effectively being denied. AI-infused practices and platforms can provide affordances for individuals’ self-governance of digital representations and for the performance of their digital identity. At the technical level, these affordances relate to the employed data types and data structures, in particular. Smith (2020) has argued that genuine, autonomous expression of identity and control over one’s digital identity is not currently compatible with the affordances for representational self-governance on social media platforms, which produce “corporatized identities” (see also Susser et al., 2019). The production of such identities primarily serves the financial interests of the corporations and, thus, constitutes treatment of individuals as means rather than ends in themselves.

Accordingly, we might distinguish between interpersonal disrespect for individuals’ informational self-determination and the sociotechnical prerequisites required for various forms of such self-governance. The former can take different forms: First, there may be non-consensual identity assignment and/or absence of possibility to self-identify (e.g., one’s gender is “predicted” by an AI system). Note also that misrepresenting the individual is *prima facie* wrong because it is untruthful (e.g., using incorrect data in making decisions concerning them), whether or not it is a violation of autonomy. Secondly, there may be a lack of meaningful alternatives for self-identification (e.g., data categories for non-binary genders may be absent in AI systems). Lastly, data may flow without individuals’ consent across agents, or individuals may have restricted control over that flow. These can be *prima facie* violations of autonomy even when sociotechnical prerequisites for informational self-governance are present. Such prerequisites might include legislation that governs data access, collection, and management, as well as material prerequisites for the effective exercise of informational self-determination, such as access to technology.

## Conclusion

This article has examined the sociotechnical bases of human autonomy (cf. Hoffmann, 2020). It has mapped various ways in which AI systems can support human autonomy or be obstacles to it. To recap, for each of the constitutive aspects of autonomy (potentials and their development, social recognition, self-respect, exercise), there are respective contrast cases (ranging from misdevelopment via misrecognition to lack of self-respect and heteronomous performance). There are also further obstacles related to the material, cultural, psychological, and informational prerequisites of autonomy. Informational considerations are not typically emphasized in general theories of human autonomy, but thanks to the nature of AI and concerns about opaqueness they merit emphasis when discussing the effects of AI on human autonomy, as pioneered by Rubel et al. (2019a), Rubel et al. (2019b), Rubel et al. (2020), Rubel et al. (2021). Typically social recognition from others and from legal institutions is directly constitutive of the relational aspect of autonomy. Recognition is at the same time a prerequisite for the development of potentials and healthy self-relations, and for the effective exercise of autonomy. Various forms of social manipulation, coercion, legal disempowerment, or political oppression are arguably the gravest obstacles to autonomy: they are direct cases of disrespect but serve at the same time as obstacles to development, exercise, or formation of positive relations to self.

An important insight, then, is that relevant ethical considerations regarding autonomy can be located in different constitutive dimensions of human autonomy. These considerations also span across different “spheres of technology experience” (Peters et al., 2018; Calvo et al., 2020), ranging from the initial adoption of a given technology to broad cultural and societal effects resulting from large-scale use. Furthermore, some issues are arguably salient equally across applications and contexts while others may arise in specific use-cases. Meaningful consent, for example, is significant in all cases, but questions regarding the appropriateness of physical interference with a person’s actions arise only with applications that have the capacity to do so (e.g., autonomous vehicles). Accordingly, analyzing the effects, AI systems have on human autonomy requires recognizing the multiplicity of its constituents, moving across levels of abstraction and spheres of technology experience, and paying careful attention to the sociotechnical context, including how AI systems interact with culture and institutions more broadly.

We further proposed a philosophical account according to which there are violations of ought-to-be norms on part of AI systems corresponding to violations of ought-to-do norms by human agents. We argued that AI systems are not moral agents and cannot have duties or literally respect or disrespect, but they are governed by so-called “ought-to-be norms”. They explain the normativity at stake with AI systems. The responsible designers, users, etc. of AI systems have ought-to-do norms, which correspond to these ought-to-be norms, in the spirit of demands for ethical design. The idea of ought-to-be norms can be held independently of the multi-dimensional, revisable model of autonomy considered throughout the study. Furthermore, we expect that this account of ought-to-be norms can be extended beyond the context of autonomy—the idea applies equally well to other topics in AI ethics, such as fairness, transparency, or privacy.

## Data Availability

The original contributions presented in the study are included in the article/Supplementary Material, and further inquiries can be directed to the corresponding author.

## Footnotes

1 As there is convincing evidence against the biological reality of human races, we use the term in scare quotes. Some human inequalities are correlated with racialized treatment, so “race” may be a social scientific category with explanatory power, and it is an ethically relevant category in virtue of past injustices.

2
Shiffrin (2000) analyzes paternalism as disrespect for agency.

3 Concerning robotic care and human dignity, this approach was introduced in Laitinen et al. (2019).

4 The content need not be restricted to violations of autonomy. Moral agents ought to also respect dignity more broadly, promote well-being, realize justice etc., and machines ought to be such that the effects on the affected parties are consistent with everyone’s autonomy, well-being, dignity, justice etc (For the central much-discussed principles, see Beauchamp and Childress, 2013. For relevant discussion in the context of AI, see Mittelstadt et al., 2016).

5 Note that different forms of violations of autonomy manifest in different ways depending on the specific AI system and use-context and can function at different levels of technology experience. Different “spheres of technology experience” (Peters et al., 2018) can function as alternative levels of abstraction for autonomy considerations and analyses (Calvo et al., 2020). First, the initial *adoption* of an AI technology itself can be based on volition and meaningful consent; conversely, consent may also be manufactured, or adoption forced. Secondly, AI *interfaces* and the *tasks* they carry out can be assessed in terms of the alternatives, choice, and control afforded to individuals, and their meaningfulness, respectively. Finally, we might consider autonomy at the level of individual and collective *behavior*, taking into account how AI systems affect individuals’ possibilities to live self-authored *lives* outside the boundaries of the technology itself, and how these effects propagate at the *societal level*. The forward- and backpropagation of technological experience across these levels (to use a metaphor fitting the context) highlights the relational dimension of autonomy in technology experience. For example, even though the social acceptance of technologies, such as self-driving vehicles, can be partly understood by looking at why and when individuals willingly adopt them, the degree of social acceptance can likewise create incentives for, or aversion to, the adoption of technology at the individual level. Rather than restricting our discussion to one specific application or level, we consider a wide range of concerns.

6 This might not be the case when it comes to algorithmic decision-making systems used by public agencies (e.g., courts) or certain private entities (e.g., companies using AI software for hiring), however.

7 Inaccuracy and bias in generalization can, of course, be problematic for reasons beyond autonomy (e.g., unfairness). Moreover, one would do well to acknowledge that respecting individuals’ right to be treated as an individual may even amount to a call for further data collection, which may be problematic once we appreciate extant problems with intrusive surveillance and uncontrolled data flows between technology companies.
